# PCV2 vaccination induces IFN-γ/TNF-α co-producing T cells with a potential role in protection

**DOI:** 10.1186/s13567-015-0157-4

**Published:** 2015-03-03

**Authors:** Hanna C Koinig, Stephanie C Talker, Maria Stadler, Andrea Ladinig, Robert Graage, Mathias Ritzmann, Isabel Hennig-Pauka, Wilhelm Gerner, Armin Saalmüller

**Affiliations:** University Clinic for Swine, Department for Farm Animals and Veterinary Public Health, University of Veterinary Medicine, Vienna, Austria; Institute of Immunology, Department of Pathobiology, University of Veterinary Medicine, Vienna, Austria; Current address: Institute of Veterinary Pathology, Vetsuisse-Faculty, University of Zurich, Zurich, Switzerland; Current address: Clinic for Swine, Ludwig-Maximilians-University, Munich, Germany

## Abstract

Porcine circovirus type 2 (PCV2) is one of the economically most important pathogens for swine production worldwide. Vaccination is a powerful tool to control porcine circovirus diseases (PCVD). However, it is not fully understood how PCV2 vaccination interacts with the porcine immune system. Especially knowledge on the cellular immune response against PCV2 is sparse. In this study we analysed antigen-specific T cell responses against PCV2 in a controlled vaccination and infection experiment. We focused on the ability of CD4^+^ T cells to produce cytokines using multicolour flow cytometry (FCM). Vaccination with a PCV2 subunit vaccine (Ingelvac CircoFLEX®) induced PCV2-specific antibodies only in five out of 12 animals. Conversely, vaccine-antigen specific CD4^+^ T cells which simultaneously produced IFN-γ and TNF-α and had a phenotype of central and effector memory T cells were detected in all vaccinated piglets. After challenge, seroconversion occurred earlier in vaccinated and infected pigs compared to the non-vaccinated, infected group. Vaccinated pigs were fully protected against viremia after subsequent challenge. Therefore, our data suggests that the induction of IFN-γ/TNF-α co-producing T cells by PCV2 vaccination may serve as a potential correlate of protection for this type of vaccine.

**Electronic supplementary material:**

The online version of this article (doi:10.1186/s13567-015-0157-4) contains supplementary material, which is available to authorized users.

## Introduction

Since the first description of porcine circovirus by Tischer et al. in 1982 [1], porcine circovirus type 2 (PCV2) has become one of the most important pathogens affecting the swine industry worldwide [2]. PCV2 is the causative agent of a number of disease syndromes summarized as porcine circovirus diseases (PCVD) among which postweaning multisystemic wasting syndrome (PMWS) is the economically most important [3,4]. Single PCV2 infection rarely results in clinical disease [5]. In the majority of cases pigs are subclinically infected [4]. However, coinfections with porcine reproductive and respiratory syndrome virus (PRRSV), porcine parvovirus (PPV) or *Mycoplasma hyopneumoniae* (*M. hyo*) are common and lead to more severe clinical symptoms [6,7].

In 2006 the first commercial PCV2 vaccines were introduced to the market [8]. Open reading frame 2 (ORF2) encoded capsid proteins were found to be immunogenic which made them suitable for vaccine development [9,10]. Indeed, two of four commercial PCV2 vaccines are based on recombinant ORF2 capsid proteins. Currently, PCV2 vaccination is widely used to combat PCVD. Different vaccines are commercially available and have successfully contributed to a decrease in mortality and an increase in growth parameters [11], probably via a reduction of PMWS severity [12]. Therefore, they are considered as an efficient tool to control PMWS [13].

The majority of domestic pigs are seropositive for PCV2-specific antibodies [12,14]. Of note, viremia is frequently detected in seropositive pigs. This led to the assumption that antibodies against PCV2 are not fully protective [15,16]. Furthermore, previous studies indicated that the analysis of antibody titres is often not sufficient to evaluate a protective immune response against PCV2 [17]. Other findings underline the importance of neutralizing antibodies. PMWS-affected pigs have lower titres of neutralizing antibodies than subclinically infected animals [18] and high titres of neutralizing antibodies are inversely correlated with PCV2 load [19]. The role of cellular immune responses for protection against PCV2 is less well studied. It was shown that cellular immune responses are directed against capsid proteins as well as against the ORF1 encoded replicase protein [20]. Apparently, CD4^+^ and CD8^+^ T cells are involved in this response [21]. In addition, one study showed that virus clearance coincides with the appearance of PCV2-specific IFN-γ secreting cells (SC) [22].

The aim of the present study was to elucidate the role of antigen-specific cellular immune responses after PCV2 vaccination with a commercially available PCV2 subunit vaccine (Ingelvac CircoFLEX®). Since the analysis of PCV2-specific antibodies is not sufficient to predict protection against PCVD, antigen-specific cellular immune responses might play an important role. During a controlled vaccination and infection experiment we monitored clinical signs and analysed PCV2 viral load in serum samples by quantitative polymerase chain reaction (qPCR). Additionally, we investigated the humoral immune response by determination of PCV2-specific antibodies using a commercially available ELISA kit. The main interest was the determination of antigen-specific T cell responses. We analysed in detail the ability of CD4^+^ T cells to produce IFN-γ and TNF-α by intracellular cytokine staining using multicolour flow cytometry (FCM). Our results provide first indications that the induction of IFN-γ/TNF-α co-producing CD4^+^ T cells by PCV2 vaccination is associated with a reduction of viremia and therefore, might contribute to a protection against PCVD.

## Materials and methods

### Animals and study design

**Figure 1:**
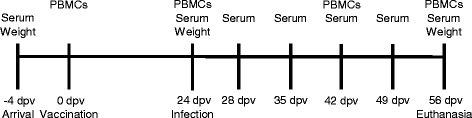
**Time schedule of the animal experiment.** Piglets were weighed after arrival and subsequently two more times in the course of the experiment. PCV2 vaccination was performed on study day 0. Piglets were inoculated with a PCV2a isolate 24 days post vaccination (dpv). Serum samples were taken twice before challenge (−4, 24 dpv) and 5 times thereafter (28, 35, 42, 49, 56 dpv). Heparinized blood samples for the isolation of PBMCs were taken on the day of vaccination, on day 24 post vaccination, 42 dpv and at the end of the study (56 dpv).

24 crossbred three week-old piglets (Large White X Landrace X Pietrain) were obtained from a conventional farm in Lower Austria and housed in the isolation unit of the Vetmeduni Vienna. On arrival, the piglets were weighed and divided into two groups according to their bodyweight. Half of the animals were vaccinated with a PCV2 subunit vaccine (Ingelvac CircoFLEX®, Boehringer Ingelheim Vetmedica, Ingelheim, Germany) according to the manufacturer’s instructions on study day 0. Twenty-four days post vaccination (dpv) half of the vaccinated and non-vaccinated pigs were intranasally infected with a PCV2a field isolate (93965, origin Poland, provided by Boehringer Ingelheim). One mL of a virus suspension containing 10^5^ TCID_50_ of PCV2/mL was administered into each nostril. During the entire experiment the pigs were kept under ordinary husbandry conditions. Infected and non-infected animals were kept in separate compartments. Clinical signs were monitored daily. Blood samples were taken by puncture of the *V. cava cranialis* or *V. jugularis* as indicated in the timeline (Figure 1). Sera were obtained for the detection of PCV2-specific antibodies and for the determination of PCV2 viremia. Whole blood samples were taken to isolate PBMCs at 0 dpv, 24 dpv, 42 dpv and 56 dpv. For calculation of the average daily weight gain, piglets were weighed three times (Figure 1). The animal experiment was approved by the institutional ethics committee, the Advisory Committee for Animal Experiments (§12 of Law for Animal Experiments, Tierversuchsgesetz – TVG) and the Federal Ministry for Science and Research (reference number BMWF 68.205/0109-II/3b/2011).

### Determination of viral load

Viremia was analysed by qPCR specific for ORF1 PCV2 DNA. The protocol for the qPCR was established at the University Clinic for Swine in cooperation with Dr Ingrid Huber (Bavarian Health and Food Safety Authority, Oberschleißheim, Germany). Both PCV2 primers and the probe attached within ORF1. Forward primer 5′-GGT ACT CCT CAA CTG CTG TCC-3′, reverse primer 5′-GGG AAA GGG TGA CGA ACT GG-3′ and the probe 5′-ACA GAA CAA TCC ACG GAG GAA GGG-3′ were purchased from TIB MOLBIOL (TIB MOLBIOL GmbH, Berlin, Germany). 6-carboxyfluorescein was used as fluorochrome and tetramethylrhodamine as quencher (TIB MOLBIOL GmbH). To create a standard curve for quantification of PCV2 DNA in the samples, a PCV2 PCR product was cloned into the PCR Cloning Vector pSC-A-amp/kan according to the manufacturer’s instructions (StrataClone^TM^ PCR Cloning Kit, Stratagene, Amsterdam, Netherlands). The insert was located in ORF1 and was produced by PCV2-specific PCR. After accumulation in *Escherichia coli* the obtained plasmid DNA was purified using Plasmid Midi Kit (Qiagen, Hilden, Germany) as recommended by the manufacturer. Different dilutions (10^2^-10^9^ copies/mL) of the purified plasmid DNA were used to establish a standard curve. As internal PCR control system a 125 bp fragment of *Nicotiana tabacum* (provided by I. Huber, Bavarian Health and Food Safety Authority) was used to avoid false negative results due to inhibitory effects of the sample matrix. Viral DNA was extracted from serum samples using High Pure PCR Template Preparation Kit (Roche, Mannheim, Germany) as recommended by the manufacturer. Thereafter DNA samples were diluted 1:10 with diethylpyrocarbonate-treated water (DEPC-treated water, Thermo Fisher Scientific, Waltham, MA, USA). A mastermix was prepared which contained Brilliant II QPCR MasterMix (Stratagene), PCV2 primers and PCV2 probe (TIB MOLBIOL GmbH) as well as primers and probe for the internal PCR control (provided by I. Huber, Bavarian Health and Food Safety Authority). The internal PCR control was added and the mastermix was pipetted to the DNA samples. A known positive serum was used as positive control. Additionally, a negative control containing DEPC-treated water (Thermo Fisher Scientific) was included in the qPCR assay. Finally, DNA fragments were amplified using Mx3005P Real Time Cycler (Stratagene). Thermocycling conditions involved an initial activation step of 95 °C for 10 min followed by 45 cycles of 95 °C for 15 s and 60 °C for 1 min. The PCR raw data was analysed using MxPro Software (Stratagene). The detection limit of the PCR was 25 virus copies/mL serum. Samples below this detection level were regarded as negative.

### Determination of PCV2-specific antibody titres

Sera were analysed for PCV2-specific antibodies by a commercially available ELISA kit (INGEZIM Circovirus IgG/IgM, Ingenasa, Madrid, Spain) according to manufacturer’s instructions. The ELISA was performed using an automated ELISA processing system (Dynex DS2®, Dynex Technologies, Chantilly, VA, USA).

### Isolation of peripheral blood mononuclear cells (PBMCs)

PBMCs were isolated from heparinized blood samples by gradient centrifugation using lymphocyte separation medium (PAA, Pasching, Austria). The isolation procedure was performed as previously described [23]. After isolation, 3–6 × 10^7^ PBMCs per cryo tube were frozen in freezing medium (50% RPMI 1640, PAN Biotech, Aidenbach, Germany, 40% fetal calf serum (FCS), PAA, 10% Dimethylsulfoxide, Sigma, Vienna, Austria) and stored at −150 °C.

### In vitro re-stimulation of PBMCs and intracellular cytokine staining (ICS)

Defrosted PBMCs were counted and 5 × 10^5^ cells per well were plated (U-bottomed 96-well microtitreplates, Greiner Bio One, Frickenhausen, Germany) in 150 μL culture medium (RPMI 1640, 10% FCS, 100 IU/mL penicillin, PAA). Cells were cultured at 37 °C and 5% CO_2_ for four hours. Subsequently, PBMCs were stimulated with 4 μg/mL baculovirus-expressed capsid protein (PCV2-ORF2, provided by Boehringer Ingelheim). Cells cultured in medium only served as negative control. Stimulation with supernatant of baculovirus infected cell cultures was performed in separate experiments and did not show any difference to the medium control. After overnight incubation, 1 μg/mL Brefeldin A (BD GolgiPlug™, BD Biosciences, San Jose, CA, USA) was added and cells were incubated for another four hours. Afterwards, 12 wells per stimulation group were pooled and PBMCs were washed twice using phosphate buffered saline (PBS, PAN Biotech) containing 3% FCS (PAA). Six-colour FCM staining was performed in U-bottomed 96-well microtitreplates (Greiner Bio One). Table 1 provides information on antibodies used for FCM staining. Prior to use, all antibodies had been titrated and optimal working dilutions were determined according to the maximal staining index. Mastermixes of antibodies were prepared for each incubation step; incubations were performed for 20 min at 4 °C in the fridge. Each incubation was followed by two washing steps with 200 μL of FCM buffer (PBS containing 3% FCS) and centrifugation. The cells were resuspended using a plate shaker after each washing step. Cell surface markers were labelled by monoclonal antibodies (mAbs) against CD4, CD8α and CD27 (see Table 1 for details), followed by incubation with isotype-specific fluorochrome-labelled secondary antibodies. Fixable Near-IR Dead Cell Stain Kit (Life Technologies, Carlsbad, CA, USA) was included in the mastermix of secondary antibodies to discriminate live from dead cells. Mastermixes for primary antibodies were prepared in PBS containing 3% FCS; secondary antibodies were prepared in PBS only. After labelling of cell surface markers, cells were fixed and permeabilized by BD Cytofix/Cytoperm (BD Biosciences) according to manufacturer’s instructions. Finally, mAbs against IFN-γ and TNF-α were added. Single stain samples for each fluorochrome were prepared as compensation controls.

**Table 1:** **Antibodies for FCM staining**

**Antigen**	**Clone**	**Isotype**	**Fluorochrome**	**Labelling strategy**	**Source of primary Ab**
CD4	74-12-4	IgG2b	Alexa488^a^	Secondary antibody	In house
CD8α	76-2-11	IgG2a	PE-Cy7^b^	Secondary antibody	In house
CD27	b30c7	IgG1	Alexa647^c^	Directly conjugated	In house
IFN-γ	P2G10	IgG1	PE^d^	Directly conjugated	BD Biosciences
TNF-α	MAb11	IgG1	BV605^e^	Directly conjugated	BioLegend

^a^Alexa488: Goat anti-Mouse IgG2b-Alexa488, Life Technologies, Carlsbad, CA, USA.

^b^PE-Cy7: Goat anti-Mouse IgG2a-PE-Cy7, Southern Biotech, Birmingham, AL, USA.

^c^Alexa647: Protein Labelling Kit Alexa 647, Life Technologies, Carlsbad, CA, USA.

^d^PE, BD Biosciences, San Jose, CA, USA.

^e^Brilliant Violet605, BioLegend, San Diego, CA, USA.

### Flow cytometry analysis

Samples were analysed using a FACSCanto II (BD Biosciences) flow cytometer equipped with three lasers (405, 488 and 633 nm). After measurement of compensation controls, compensation was calculated by FACSDiva software Version 6.1.3 (BD Biosciences). Flow cytometric data was analysed with the same version of FACSDiva software.

### Statistics and preparation of diagrams

Statistics were calculated using IBM SPSS® Statistics 20 (IBM Corp., Armonk, NY, USA). Data was tested for normal distribution using Kolmogorov-Smirnov test. Analysis of variance (ANOVA) was performed to compare the four study groups. Tukey’s range test was used for post-hoc analysis. For not normally distributed data Kruskal-Wallis test and consequently Mann–Whitney tests were executed to evaluate differences between the treatment groups. To analyse differences within one study group, paired t-tests were conducted for normally distributed data and Wilcoxon tests were used for not normally distributed data. Bar charts and scatter diagrams were created in Excel 2010 (Microsoft, Redmond, WA, USA).

## Results

To evaluate the influence of vaccination against PCV2 with the recombinant baculovirus PCV2-ORF2 vaccine (Ingelvac CircoFLEX®) and subsequent challenge on the generation of antigen-specific T cells, 24 piglets were divided into four groups. Six piglets were immunized intramuscularly with the Ingelvac CircoFLEX® vaccine and represented the vaccinated group (VA). Another six piglets were vaccinated and intranasally infected (VI) at 24 dpv with a PCV2a isolate. An additional group of six animals was non-vaccinated and infected with the same virus strain at day 24 and represented the infection group (IN). Six non-vaccinated and non-infected animals served as controls (CO). The time course of the experiment is presented in Figure 1.

**Figure 2:**
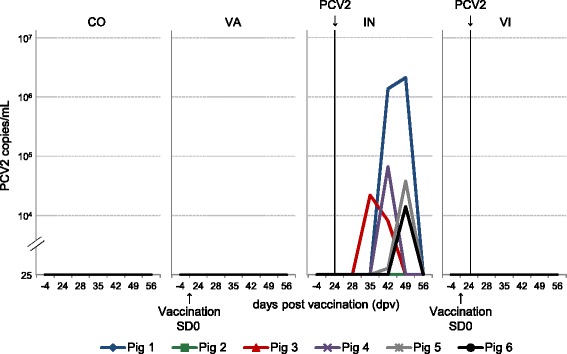
**Viral load in animals of the different treatment groups.** Sera were analysed for PCV2 DNA by quantitative PCR specific for ORF1. Line charts represent levels of virus titres in serum samples of individual animals within the four study groups (CO = control, VA = vaccinated, IN = infected, VI = vaccinated & infected) at all investigated time points before and after PCV2 infection. The detection level of the qPCR was 25 virus copies/mL serum. Samples below this level were regarded as negative. The arrows indicate PCV2 vaccination on study day 0 (SD0) and experimental PCV2 infection on day 24 post vaccination, respectively.

### Clinical signs

On arrival, all piglets included in the study were clinically healthy. Vaccination did not induce any clinical signs. Likewise, no signs of disease were detected after PCV2 inoculation. None of the piglets showed any signs of PCVD throughout the entire study, despite the fact that PCV2 DNA could be detected in serum of five out of six animals in the IN group starting from day 35 (see below). No significant difference was found in average daily weight gain before and after PCV2 inoculation between the four study groups (data not shown).

### PCV2 viral load in serum samples

**Figure 3:**
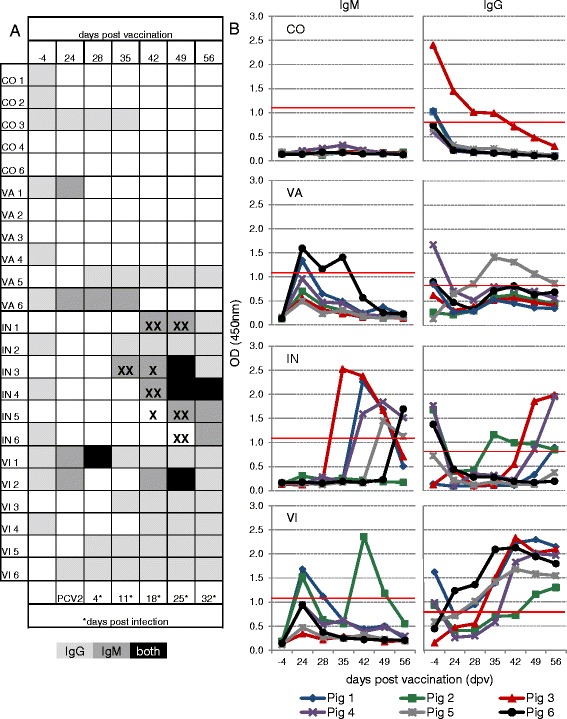
**PCV2-specific IgM/IgG antibodies and coincidence with viremia.** Serum samples were analysed for PCV2-specific antibodies by a commercially available ELISA kit (INGEZIM Circovirus IgG/IgM, Ingenasa, Madrid, Spain). **A)** The heatmap shows the detection of PCV2-specific IgM and IgG antibodies for all animals of the different study groups (CO = control, VA = vaccinated, IN = infected, VI = vaccinated & infected). Light grey fields represent samples positive for IgG. Dark grey fields indicate the detection of IgM antibodies. Black fields illustrate samples containing both IgM and IgG antibodies. PCV2 infection was performed 24 days post vaccination. Corresponding days post infection are indicated at the bottom of the table. Occurrence of PCV2 DNA in individual samples is marked by X. X indicates serum samples with < 10^4^ PCV2 copies/mL, XX stands for > 10^4^ PCV2 copies/mL. **B)** Raw data of the INGEZIM Circovirus IgG/IgM ELISA is depicted. OD-values, measured at 450 nm, of IgM (left) and IgG antibodies (right) for individual animals and all sampling points are shown. The red lines indicate the cut-off OD = 1.108 for IgM and OD = 0.821 for IgG, respectively.

Sera of all animals were investigated by qPCR for ORF1 DNA. No PCV2 nucleic acids were found in the serum samples taken on study day −4 and 24 dpv, i.e. prior to infection, and also four days after infection (28 dpv). The earliest time point for the detection of PCV2 DNA in the sera was 35 dpv (11 days after infection), and only one animal in the IN group was positive (Figure 2, IN, animal #3, IN3). In IN animals viremia could be detected at 42 dpv (18 days after infection) in four out of six animals. A viral load of > 10^4^ PCV2 copies/mL serum was found in four of the positive pigs (IN3, IN4, IN5, IN6). One animal (IN1) had a viral load of >10^6^ PCV2 copies/mL on two consecutive examination time points (42 dpv, 49 dpv) (Figure 2, IN). Another animal (IN2) did not show any detectable viremia at all investigated time points after infection. At 56 dpv (32 days after infection) no viral DNA could be detected in any sera of the IN group. All animals in the VI group (Figure 2) stayed negative for PCV2 DNA during the entire experiment. Likewise, sera of the CO and VA group remained free of PCV2 nucleic acids (Figure 2 and summarized in Figure 3A).

### Serology

For a determination of the humoral immune response after PCV2 vaccination and infection, we analysed serum samples by a commercial ELISA (INGEZIM Circovirus IgG/IgM, Ingenasa, Madrid, Spain) which differentiates between PCV2-specific IgM and IgG antibodies. Results are displayed in a heat map, indicating the presence of PCV2-specific IgM and IgG antibodies, or both (Figure 3A). In addition, OD-values derived from this ELISA are shown in Figure 3B to allow a semi-quantitative evaluation of the titres in individual animals.

Due to the parallel IgM/ IgG detection, early after birth the test potentially discriminates between maternal PCV2-specific IgG antibodies and PCV2-specific IgM antibodies produced by the piglet. At the beginning of the experiment at day −4, twelve out of 24 piglets had PCV2-specific IgG antibodies, which were most likely maternally derived antibodies (Figures 3A and B). However, at 24 dpv only two animals were positive for IgG, indicating a decline of maternal antibodies. At this time point (24 dpv), within vaccinated animals (VA, VI), four were positive for IgM which most probably were induced by vaccination and one was positive for IgG. Of note, although titres remained below the cut-off value, in tendency all vaccinated animals showed an increase in OD-values for IgM from −4 dpv to 24 dpv (Figure 3B, VA, VI). After PCV2 inoculation (day 24), piglets from the vaccinated group (Figures 3A and B, VI) showed an earlier seroconversion than piglets from the IN group. Four days after infection (28 dpv), three out of six VI piglets had PCV2-specific antibodies. One animal (Figures 3A and B, VI1) showed IgG and IgM antibodies, whereas two other animals (VI5 and VI6) had IgG only. At 35 dpv four out of six animals in the VI group had seroconverted; 18 days after infection (42 dpv) all animals of the VI group showed a detectable antibody titre, with IgG antibodies being present in five of out of six animals. Only one animal (VI2) had IgM antibodies which could still be detected seven days later (49 dpv) together with IgG molecules. Another seven days later (56 dpv) only IgG could be identified in piglet VI2. PCV2-specific antibodies developed later in IN animals (Figures 3A and B, IN). Four days after infection (28 dpv), all piglets were negative. Seven days later (35 dpv) two out of six animals showed PCV2-specific antibodies, IN2 IgG and IN3 IgM. The IgM antibodies in IN3 coincided with viremia (Figure 3A, IN, black crosses). Another seven days later (18 days after infection, 42 dpv) IgM antibodies were present in three animals, all with a detectable viremia (IN1, IN3, IN4). One animal with detectable virus DNA (IN5) had not yet seroconverted and one animal without detectable viral DNA showed seroconversion to IgG antibodies (IN2). At 49 dpv individual animals of the IN group had either IgG, IgM or both types of antibodies and 56 dpv all IN animals were seropositive. Unlike VI animals, which were predominantly IgG positive, antibodies of both investigated isotypes were found across animals of the IN group. In contrast to the infected groups (IN, VI), pigs of the VA and CO group were seronegative at the three time points at the end of the experiment (42 dpv, 49 dpv, and 56 dpv), with the exception of one VA animal (Figure 3, VA5). Nevertheless, although titres remained below the cut-off value, in tendency all VA animals showed an increase in OD-values for IgG from 35 dpv to 56 dpv (Figure 3B).

### Cytokine production of CD4^+^ T cells and phenotype of cytokine-producing cells

**Figure 4:**
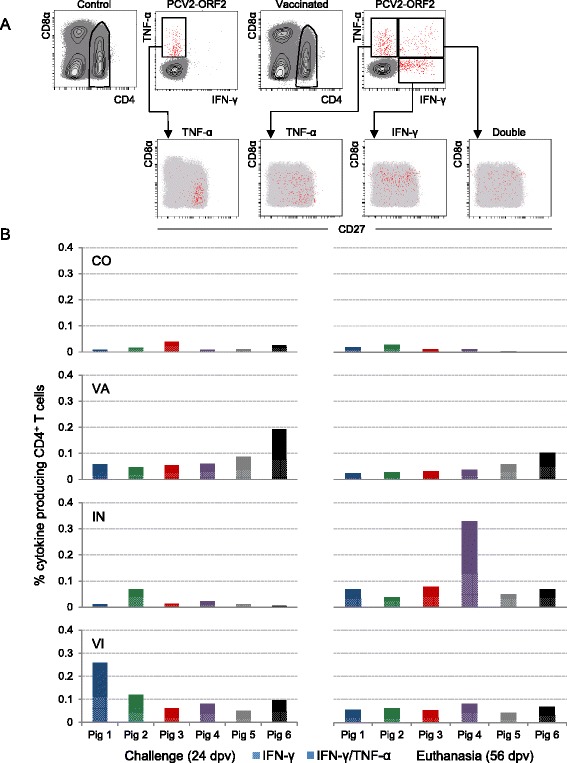
**Frequency of PCV2-ORF2-specific cytokine-producing CD4** ^**+**^ **T cells. A)** Porcine PBMCs were stimulated with PCV2-ORF2 over night or were cultured in medium as negative control. CD4^+^ T cells were gated and analysed for production of IFN-γ and TNF-α by intracellular cytokine staining (top panel). Cytokine-producing CD4^+^ T cell subsets were further sub-gated for the analysis of CD8α and CD27 expression (bottom panel). Red dots indicate cytokine-producing CD4^+^ T cells; grey dots represent total CD4^+^ T cells. FCM data is shown for one representative animal of the control (CO) and vaccinated group (VA) at 24 dpv. **B)** Stacked bar charts indicate percentages of cytokine-producing CD4^+^ T cells within total CD4^+^ T cells from individual animals of the treatment groups (CO = control, VA = vaccinated, IN = infected, VI = vaccinated & infected) prior to infection (24 dpv) and at the end of the study (56 dpv). Hatched bars represent percentages of IFN-γ single-producing CD4^+^ T cells and filled bars illustrate percentages of IFN-γ/TNF-α co-producing CD4^+^ T cells within total CD4^+^ T cells.

The main focus of this study was the analysis of PCV2-specific CD4^+^ T cell responses in regard to IFN-γ/TNF-α production and phenotyping of responding cells. PBMCs were re-stimulated with the vaccine antigen (PCV2-ORF2) in vitro and analysed by multicolour FCM. Cells cultured in medium alone served as controls. In order to identify rare subsets of cytokine-producing T cells, at least 8 × 10^5^ lymphocytes per sample, identified by light scatter properties, were acquired during FCM analyses. Total CD4^+^ T cells were gated (Figure 4A) and analysed for IFN-γ and TNF-α production. To evaluate the memory phenotype of cytokine-producing T cells we further analysed the expression of the surface markers CD8α and CD27. The gating strategy and the cytokine analysis are displayed from representative experiments with PBMCs isolated from a control (CO, left) and a vaccinated (VA, right) animal in Figure 4A. Hardly any IFN-γ and IFN-γ/TNF-α co-producing T cells were detected in pigs belonging to the CO group. In contrast, TNF-α single production in response to PCV2-ORF2 was also found in CO animals. These TNF-α single-producing T cells from CO animals were CD4^+^ and mostly had a naïve phenotype (CD8α^−^), indicating that this subpopulation does not represent PCV2-specific memory cells. Therefore, TNF-α single-producing T cells were excluded from further analyses. In contrast, in the vaccinated groups (VA, VI) IFN-γ single-producing as well as IFN-γ/TNF-α co-producing T cells were induced after vaccination. These cells were mainly CD8α^+^ and therefore, considered as antigen experienced. CD27^+^ central memory T cells (T_CM_) and CD27^−^ effector memory T cells (T_EM_) were present in this population of cytokine-producing T cells (Figure 4A).

Percentages of CD4^+^ IFN-γ single and IFN-γ/TNF-α co-producing T cells at two investigated time points, prior to challenge (24 dpv) and at the end of the study (56 dpv), for the individual animals within respective groups are shown in Figure 4B. Moreover, the mean values of these two subpopulations for each treatment group were calculated and subjected to statistical analyses (Additional file 1). Background levels of about 0.02% IFN-γ single-producing cells within total CD4^+^ T cells were identified in PBMCs isolated from CO animals at 24 dpv and at the end of the study (56 dpv) and the frequencies of IFN-γ/TNF-α co-producing T cells were even lower (≤0.014%). Cytokine production prior to vaccination was tested for three animals belonging to either the VI or the VA group. At 0 dpv CD4^+^ T cells of all animals showed minimal IFN-γ/TNF-α production (IFN-γ^+^TNF-α^−^ ≤ 0.014%, IFN-γ^+^TNF-α^+^ ≤ 0.0098%; Additional file 2). Also, cytokine production in samples incubated with cell culture medium only was close to zero for all animals and time points (data not shown).

Compared to the CO group the frequency of IFN-γ producing T cells isolated from animals of the VA group was higher at 24 dpv. By this time, all animals of the VA group had higher levels of IFN-γ/TNF-α co-producing T cells than the controls (*p* = 0.044). Animal VA6 showed the most prominent response to vaccination, especially regarding the development of double cytokine-producing T cells. The frequency of IFN-γ single as well as IFN-γ/TNF-α co-producing T cells declined in all VA animals (*p* = 0.004 for IFN-γ and p = 0.028 for IFN-γ/TNF-α co-producing T cells, respectively) towards the end of the study (56 dpv).

Before challenge, the IN pigs had similar low frequencies of IFN-γ^+^ and IFN-γ^+^TNF-α^+^ T cells as animals from the CO group. After experimental PCV2 infection, we observed a strong increase of IFN-γ single-producing T cells in this group. IN4 had the strongest response to PCV2 infection with a 15-fold enhancement of IFN-γ^+^CD4^+^ T cells by the end of the study. Similar results were obtained for IFN-γ/TNF-α co-producing T cells (*p* = 0.046). We found a strong increase of double cytokine-producing T cells after PCV2 infection in five IN pigs, among which IN4 was outstanding (0.1996%). IN2 had low levels (0.03%) of double cytokine-producing T cells before challenge which did not further increase after PCV2 infection.

Similar to VA animals, VI pigs had elevated frequencies of IFN-γ producing CD4^+^ T cells compared to their non-vaccinated counterparts at 24 dpv. Furthermore, a clear induction of bi-functional T cells in response to vaccination was detected in all VI animals 24 dpv. In contrast to IN pigs, no further increase of single or double cytokine-producing CD4^+^ T cells was observed after PCV2 infection. Instead, the frequency of cytokine-producing T cells slightly declined towards the end of the study.

**Figure 5:**
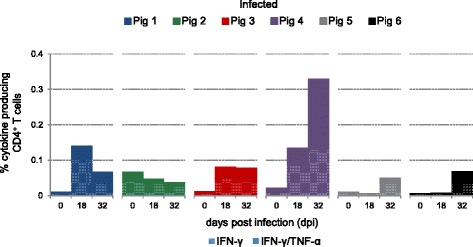
**Kinetics of PCV2-specific cytokine-producing CD4** ^**+**^ **T cells following PCV2 infection.** PBMCs isolated from PCV2 infected animals (not vaccinated, IN-group) before infection and 18 or 32 days post infection (dpi) were stimulated with PCV2-ORF2. CD4^+^ T cells were gated as described before and analysed for IFN-γ and TNF-α production. Stacked bar charts indicate percentages of cytokine-producing CD4^+^ T cells from individual animals. Hatched bars represent percentages of IFN-γ single-producing CD4^+^ T cells and filled bars illustrate percentages of IFN-γ/TNF-α co-producing CD4^+^ T cells within total CD4^+^ T cells.

Results from the VI group had indicated that an increase in IFN-γ single and IFN-γ/TNF-α double-producing CD4^+^ T cells led to an accelerated occurrence of PCV2-specific IgG antibodies following PCV2 infection. This raised the question if the IgG antibodies seen at 32 days post infection (dpi) in four out six animals of the IN group were also preceded by an increase of PCV2-specific cytokine-producing CD4^+^ T cells. Therefore, PBMCs isolated from the IN group were tested also 18 dpi and data is displayed in a time course (Figure 5). Indeed, three of the four animals with PCV2-specific IgG antibodies at 32 dpi (IN1, IN3, IN4) showed a strong increase of IFN-γ single and IFN-γ/TNF-α double-producing CD4^+^ T cells at 18 dpi. IN5 and IN6 who lacked single and double cytokine-producing T cells 18 dpi were only positive for IgM by the end of the study and did not perform a class switch to IgG.

**Figure 6:**
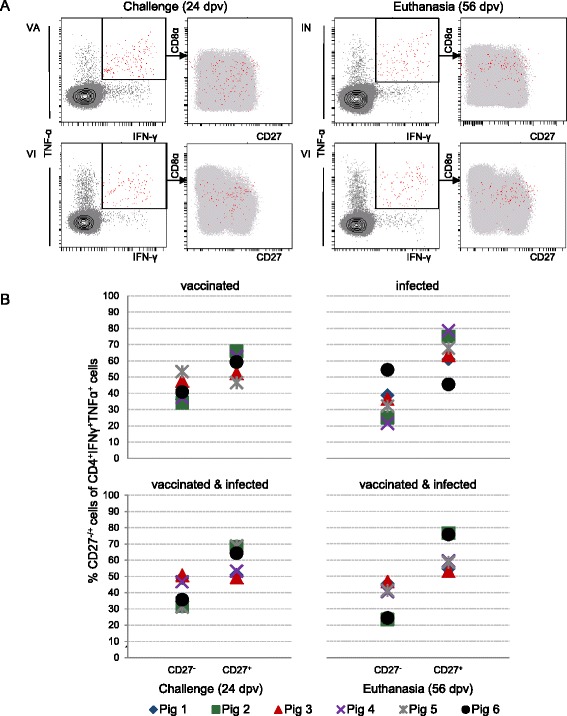
**Memory phenotype of IFN-γ/TNF-α co-producing CD4** ^**+**^ **T cells. A)** CD4^+^ T cells were gated (not shown) and analysed for IFN-γ and TNF-α production following PCV2-ORF2 restimulation. IFN-γ/TNF-α double cytokine-producing CD4^+^ T cells were sub-gated and investigated for CD8α and CD27 expression. Representative data of animals VA6 (24 dpv), IN3 (56 dpv) and VI6 (challenge and euthanasia) are shown. **B)** Symbols in the scatter diagrams represent percentages of CD27^−^ effector memory T cells (T_EM_) and CD27^+^ central memory T cells (T_CM_) within the subpopulation of IFN-γ/TNF-α co-producing T cells. Data is shown for individual animals of the vaccinated (VA), infected (IN) and vaccinated & infected (VI) group. The investigated time points correspond to 24 dpv (challenge) and 56 dpv (euthanasia).

Lastly, having observed the marked induction of IFN-γ/TNF-α co-producing CD4^+^ T cells following PCV2 vaccination and also PCV2 infection, we compared the CD27 expression in PCV2-specific IFN-γ^+^TNF-α^+^CD4^+^ T cells from VA animals at 24 dpv (day of experimental infection), IN animals at the day of euthanasia and VI animals at both time points (Figure 6). Regardless of the treatment group and the time point, CD27^+^ central memory T cells (T_CM_) were present in higher frequencies than CD27^−^ effector memory T cells (T_EM_) in the vast majority of samples.

## Discussion

PCV2 continues to be a highly relevant pathogen for modern swine production which needs to be controlled by vaccination [2]. The role of the humoral immune response after PCV2 vaccination has been extensively studied but knowledge on cellular immune responses is sparse. Based on a controlled PCV2 vaccination-infection experiment, the present study puts a strong focus on the role of CD4^+^ T cells and their potential role following vaccination and/ or infection.

### Clinical signs

Clinical manifestation of PCV2 infection varies and can result in distinct diseases. Most pigs are subclinically infected. In other cases lung disease, enteric disease, reproductive failure or porcine dermatitis and nephropathy syndrome (PDNS) may occur. PMWS is the economically most important manifestation of PCV2 infection [3,4]. It is generally accepted, that PCV2 alone is not sufficient to induce PCVD. Coinfections with other viral or bacterial pathogens are needed to provoke more severe clinical symptoms [5]. Accordingly, we did not observe any signs of PCVD after singular PCV2 infection in our animal experiment.

### PCV2 viral load in serum samples

Detection of PCV2 antigen within characteristic microscopic lesions is one of the key requirements for PCVD diagnosis [24,25]. In addition, determination of high viral loads (> 10^4.7^ PCV2 copies/mL) in serum samples by qPCR is an indicator for PCVD [4]. Hence, we analysed serum samples collected at regular intervals throughout the vaccination-infection experiment for the amount of PCV2 DNA. In our study, viremia occurred in the IN group only. Interestingly, piglets vaccinated with the PCV2 subunit vaccine seemed to be protected and did not develop viremia after PCV2 inoculation (Figure 2). This is in accordance with other studies: in field studies, the same PCV2 subunit vaccine significantly reduced PCV2 viral load and duration of viremia in sera of pigs suffering from PMWS and porcine respiratory disease complex (PRDC), respectively [26,27]. Under experimental conditions, four commercially available PCV2 vaccines, including the subunit vaccine used in our study, significantly decreased PCV2 DNA in the blood after PCV2 challenge [28]. However, in all these studies viremia was not completely prevented by PCV2 vaccination. Nevertheless, we assume that our experimental infection was successful because similar levels of viremia were achieved after singular PCV2 infection elsewhere [28].

### Serology

Serum antibodies against PCV2 are wide spread in pig populations all over the world [12,14]. However, the analysis of PCV2-specific antibody titres is challenging from a technical perspective. Several studies revealed that vast differences among laboratories and various ELISA test systems exist [29,30]. Despite these issues, we decided to use a qualitative ELISA capable of distinguishing IgM from IgG antibodies. This enabled us to differentiate between maternally derived and vaccine induced antibodies. 24 dpv PCV2-specific antibodies, which were considered to be induced by vaccination, were detected in five out of 12 vaccinated piglets. After experimental infection, all infected animals seroconverted independent of vaccination status. Of note, seroconversion occurred earlier in VI pigs than in IN pigs. These results are comparable to previous data. Several recent studies described that pigs vaccinated with PCV2 subunit vaccines showed a prompt seroconversion and had increased antibody titres compared to their non-vaccinated counterparts under experimental conditions as well as after natural PCV2 infection [28,31].

Although all animals of the VI group had detectable PCV2-specific IgG titres at the end of the study, for some individuals this was not preceded by a clear IgM response. However, although titres remained below the cut-off value, in tendency also VI animals 3, 4, 5 and 6 showed an increase in OD-values for IgM from −4 dpv to 24 dpv. In addition, it is conceivable that these animals with IgM titres below the cut-off had higher titres before day 24 post vaccination. Nevertheless, antibodies of the IgG isotype were predominant in samples of VI pigs, whereas a mixture of IgM and IgG antibodies was found in IN pigs (Figure 3). The early occurrence of IgG antibodies is most probably a beneficial effect of the vaccination which may be related to the presence of vaccine-specific CD4^+^ T cells with a helper function (see also below).

### Cytokine production of CD4^+^ T cells and phenotype of cytokine-producing cells

It is likely that a sufficient protection against PCV2 infection is achieved by a response of both the humoral and the cellular immune system following vaccination. So far, little is known about the cellular immune response and knowledge on the development of antigen-specific T memory cells after PCV2 vaccination is sparse. Therefore, a detailed examination of vaccine induced antigen-specific CD4^+^ T cells and their IFN-γ/TNF-α production was the main focus of our study. To evaluate the memory phenotype of cytokine-producing cells, we further analysed the expression of CD8α and CD27. As mentioned above, CD8α can be found on activated, antigen experienced T cells and memory T cells whereas naïve T helper cells are considered CD8α negative [32]. Similar to humans CD27 distinguishes between porcine central and effector memory T cells [33]. IFN-γ single and IFN-γ/TNF-α co-producing CD4^+^ T cells induced after vaccination were mainly activated CD8α^+^ T cells. We detected a homogenous distribution of CD27^+^ central memory T cells (T_CM_) and CD27^−^ effector memory T cells (T_EM_) within IFN-γ/TNF-α co-producing T cells in VA, IN and VI pigs. Apparently, all memory phenotypes were addressed equally by PCV2 vaccination and/ or infection (Figure 4A and Figure 6). With these findings we expand previous knowledge that PCV2 vaccination elicits CD4^+^CD8α^+^ memory T cells [34].

So far, it is known that the development of IFN-γ secreting cells (SC) is a key event in cell-mediated PCV2 immunity [35]. A good example for that is the inverse correlation of IFN-γ SC and numbers of PCV2 copies in serum samples [28,36]. In the present study, we additionally investigated the phenotype of the IFN-γ producing T cells by ICS after in vitro re-stimulation with PCV2-ORF2. We detected a higher frequency of IFN-γ single-producing CD4^+^ T cells in vaccinated animals compared to their non-vaccinated counterparts at 24 dpv. Similarly, the frequency of IFN-γ producing CD4^+^ T cells increased in the IN group after experimental PCV2 infection (Figure 4B, Figure 5). Obviously, this increase of antigen-specific IFN-γ producing cells is a response to vaccination and infection. Similar results were obtained in studies investigating cell-mediated immune responses against PCV2 based on determination of IFN-γ SC by ELISpot assay. In accordance with our data, IFN-γ SC in response to capsid protein were induced in vaccinated as well as in vaccinated and challenged pigs [37]. Amounts of PCV2-specific IFN-γ SC increased also in vaccinated and naturally PCV2 infected pigs [38]. Unlike others, we did not detect a further enhancement of cytokine-producing cells after experimental infection in vaccinated and infected pigs [39].

Evidence exists that the magnitude of IFN-γ production by CD4^+^ T cells alone is not always sufficient to predict protection [40] and that multi-functional T cells, which simultaneously produce IFN-γ, interleukin-2 (IL-2) and TNF-α, are strongly correlated with protection [41]. Hence, in addition to IFN-γ, we investigated TNF-α expression by CD4^+^ T cells and consequently evaluated the appearance of IFN-γ/TNF-α co-producing T cells. 24 dpv we found elevated levels of IFN-γ^+^TNF-α^+^CD4^+^ T cells in all vaccinated animals (VA, VI). The frequency of these cells declined in the VA group towards the end of the study (56 dpv). In the IN group we also observed these double cytokine-producing T cells following PCV2 inoculation. Similar to IFN-γ single-producing cells, no further increase of co-producing T cells was observed in the VI group after PCV2 challenge (Figure 4B). Nevertheless, it is conceivable that the induction of PCV2-specific IFN-γ/TNF-α co-producing CD4^+^ T cells by a non-replicative agent, i.e. the ORF2 subunit vaccine, is sufficient to control a subsequent PCV2 infection. Another important finding of this study is that the occurrence of cytokine-producing CD4^+^ T cells 18 dpi was associated with a subsequent isotype-switch from IgM to IgG in the IN group. This phenomenon may be interpreted as a helper function of CD4^+^ T cells for antibody production and antibody class switch and support the hypothesis that these double cytokine-producing T cells play a role in protection against PCV2.

In conclusion, our data shows that PCV2 vaccination did not induce stable titres of PCV2-specific IgG antibodies in all vaccinated animals. Nevertheless, the PCV2 subunit vaccine was highly efficacious and prevented viremia in all vaccinated and infected pigs. Moreover, the amount of IFN-γ producing CD4^+^ T cells increased after PCV2 vaccination and/ or infection. Contrary to PCV2-specific antibodies, antigen-specific IFN-γ^+^TNF-α^+^CD4^+^ T cells were induced in all vaccinated pigs. The appearance of these cells seems to be linked to a switch from IgM to IgG indicating their relevance for protection after PCV2 infection. Therefore, IFN-γ/TNF-α co-producing T cells might represent a new correlate of an immune reaction after PCV2 vaccination and their existence after vaccination might correlate with protection. This finding needs further investigation.

## Additional files

Additional file 1:
**Mean cytokine production of PCV2-ORF2 specific CD4** ^**+**^ **T cells.** Stacked bar charts indicate mean percentages of cytokine-producing CD4^+^ T cells of the respective groups (CO = control, VA = vaccinated, IN = infected, VI = vaccinated & infected) at the investigated time points prior to challenge (C, 24 days post vaccination) and at the day of euthanasia (E, 56 days post vaccination). Hatched bars represent percentages of IFN-γ single-producing CD4^+^ T cells and filled bars illustrate percentages of IFN-γ/TNF-α co-producing CD4^+^ T cells. Minuscules indicate significant differences (*p* < 0.05) between treatment groups and investigated time points, respectively. For IFN-γ single-producing CD4^+^ T cells significant differences were detected between CO and IN at the end of the study (E) (a:b *p* = 0.035) and within the VA group between 24 dpv (C) and 56 dpv (E) (c:d *p* = 0.004). For IFN-γ/TNF-α co-producing CD4^+^ T cells differences were significant on day 24 post vaccination between CO and VA (e:f *p* = 0.044), CO and VI (e:g *p* = 0.006) and IN and VI (g:h *p* = 0.011). On study day 56 post vaccination differences were significant between CO and VA, IN and VI, respectively (i:j *p* = 0.006). Within the IN group more IFN-γ/TNF-α co-producing CD4^+^ T cells were detected after experimental infection (h:j *p* = 0.046) and in both vaccinated groups (VA and VI) frequency of double cytokine-producing cells declined towards the end of the study (56 dpv) (f:j; g:j *p* = 0.028). Additional file 2:
**Cytokine production of CD4** ^**+**^ **T cells in the time course before and after PCV2 vaccination.** The frequency of PCV2-ORF2-specific cytokine-producing CD4^+^ T cells in the time course before and after vaccination for three animals of the vaccinated (VA, top panel) and three animals of the vaccinated and infected group (VI, bottom panel) was analysed. Contour plots display data from one representative animal (VI1) before vaccination and 24 days post vaccination (dpv). PBMCs were stimulated with PCV2-ORF2 and CD4^+^ T cells were gated as described above. Stacked bar charts indicate percentages of cytokine-producing CD4^+^ T cells before vaccination as well as 24 and 56 dpv from individual animals. Hatched bars represent percentages of IFN-γ single-producing CD4^+^ T cells and filled bars illustrate percentages of IFN-γ/TNF-α co-producing CD4^+^ T cells within total CD4^+^ T cells.

## Abbreviations

BV: Brilliant violet
CD: Cluster of differentiation
CO: Control group
DMSO: Dimethyl sulfoxide
dpi: Days post infection
dpv: Days post vaccination
ELISA: Enzyme linked immunosorbent assay
ELISpot: Enzyme linked immunosorbent spot
FCM: Flow cytometry
FCS: Fetal calf serum
ICS: Intracellular cytokine staining
IFN-γ: Interferon gamma
IL-2: Interleukin 2
IN: Infection group
IU: International unit
mAB: Monoclonal antibody
*M. hyo*: *Mycoplasma hyopneumoniae*
ORF: Open reading frame
PBMCs: Peripheral blood mononuclear cells
PBS: Phosphate buffered saline
PCV2: Porcine circovirus type 2
PCVD: Porcine circovirus diseases
PDNS: Porcine dermatitis and nephropathy syndrome
PE: Phycoerythrin
PMWS: Postweaning multisystemic wasting syndrome
PPV: Porcine parvovirus
PRRSV: Porcine reproductive and respiratory syndrome virus
qPCR: Quantitative polymerase chain reaction
SC: Secreting cells
T_CM_: Central memory T cells
T_EM_: Effector memory T cells
TNF-α: Tumor necrosis factor alpha
VA: Vaccination group
VI: Vaccinated and infected group
