# Supplementary figures and images for: PCV2 vaccination induces IFN-γ/TNF-α co-producing T cells with a potential role in protection
Source: Vet Res. 2015 Mar 3;46:20. doi: 10.1186/s13567-015-0157-4 (PMC4348102; doi:10.1186/s13567-015-0157-4)

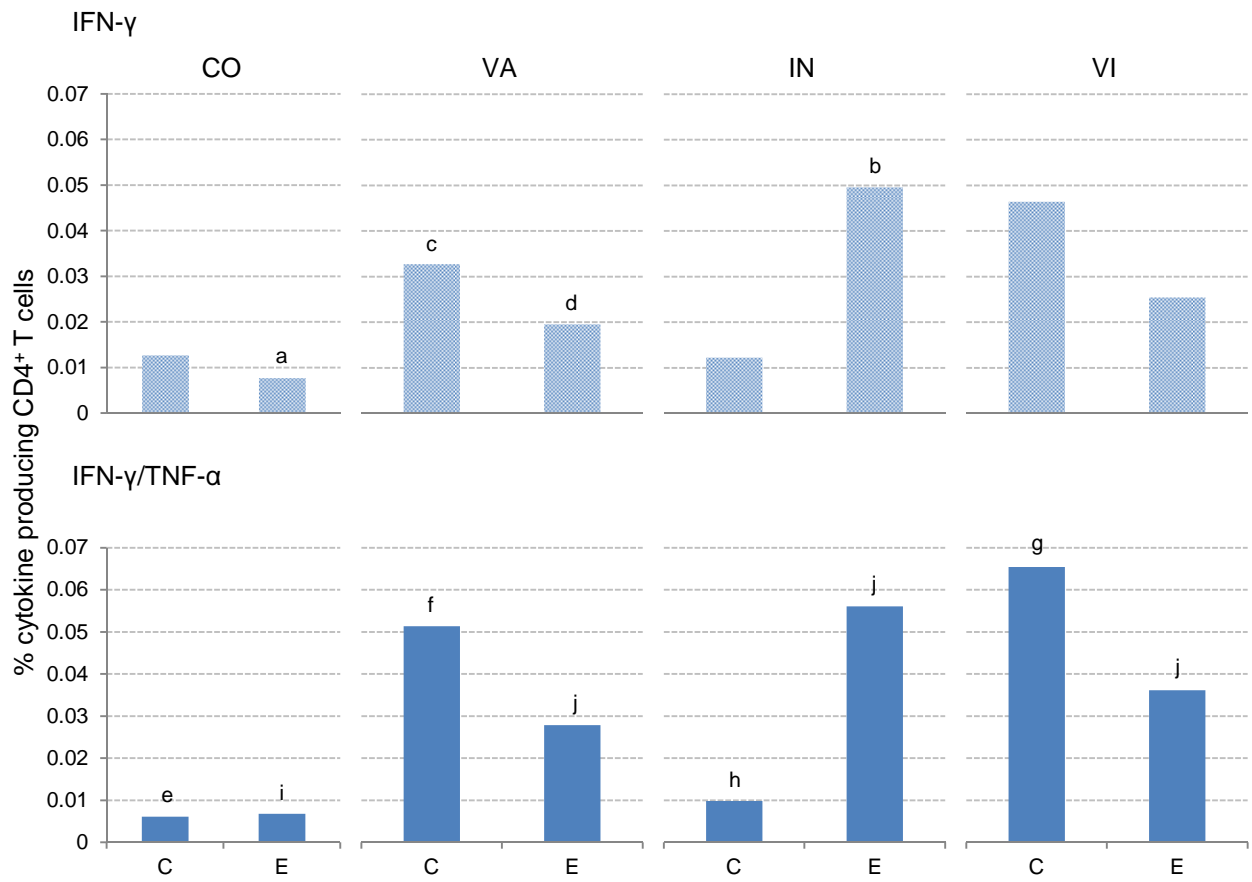

Supplement: Additional file 1: — Mean cytokine production of PCV2-ORF2 specific CD4 + T cells. Stacked bar charts indicate mean percentages of cytokine-producing CD4+ T cells of the respective groups (CO = control, VA = vaccinated, IN = infected, VI = vaccinated & infected) at the investigated time points prior to challenge (C, 24 days post vaccination) and at the day of euthanasia (E, 56 days post vaccination). Hatched bars represent percentages of IFN-γ single-producing CD4+ T cells and filled bars illustrate percentages of IFN-γ/TNF-α co-producing CD4+ T cells. Minuscules indicate significant differences (p < 0.05) between treatment groups and investigated time points, respectively. For IFN-γ single-producing CD4+ T cells significant differences were detected between CO and IN at the end of the study (E) (a:b p = 0.035) and within the VA group between 24 dpv (C) and 56 dpv (E) (c:d p = 0.004). For IFN-γ/TNF-α co-producing CD4+ T cells differences were significant on day 24 post vaccination between CO and VA (e:f p = 0.044), CO and VI (e:g p = 0.006) and IN and VI (g:h p = 0.011). On study day 56 post vaccination differences were significant between CO and VA, IN and VI, respectively (i:j p = 0.006). Within the IN group more IFN-γ/TNF-α co-producing CD4+ T cells were detected after experimental infection (h:j p = 0.046) and in both vaccinated groups (VA and VI) frequency of double cytokine-producing cells declined towards the end of the study (56 dpv) (f:j; g:j p = 0.028). [file 13567_2015_157_MOESM1_ESM.pdf]

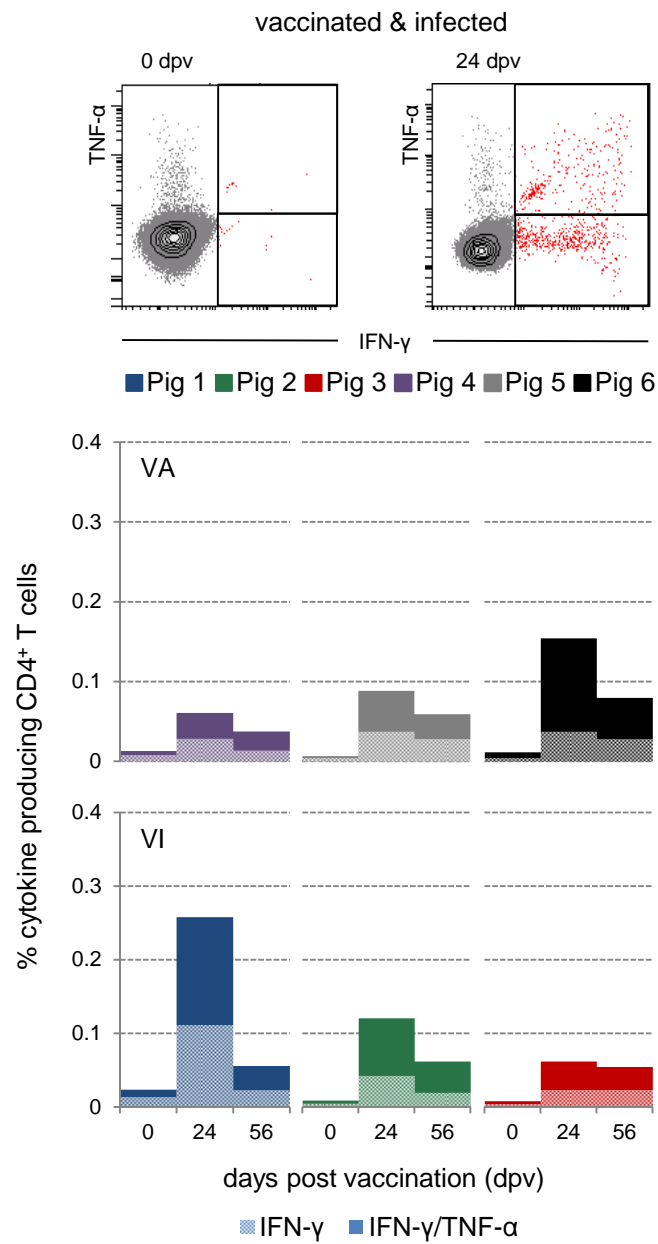

Supplement: Additional file 2: — Cytokine production of CD4 + T cells in the time course before and after PCV2 vaccination. The frequency of PCV2-ORF2-specific cytokine-producing CD4+ T cells in the time course before and after vaccination for three animals of the vaccinated (VA, top panel) and three animals of the vaccinated and infected group (VI, bottom panel) was analysed. Contour plots display data from one representative animal (VI1) before vaccination and 24 days post vaccination (dpv). PBMCs were stimulated with PCV2-ORF2 and CD4+ T cells were gated as described above. Stacked bar charts indicate percentages of cytokine-producing CD4+ T cells before vaccination as well as 24 and 56 dpv from individual animals. Hatched bars represent percentages of IFN-γ single-producing CD4+ T cells and filled bars illustrate percentages of IFN-γ/TNF-α co-producing CD4+ T cells within total CD4+ T cells. [file 13567_2015_157_MOESM2_ESM.pdf]
